# Non-coding RNAs at the intersection of epigenetics and cancer metabolism

**DOI:** 10.3389/freae.2025.1699969

**Published:** 2025-10-20

**Authors:** Nicolò Mangraviti, Serena Castelli

**Affiliations:** ^1^ Department of Neuroscience and Physiology, NYU Grossman School of Medicine, NYU Langone Health, New York, NY, United States; ^2^ Department for the Promotion of Human Science and Quality of Life, San Raffaele Open University, Rome, Italy

**Keywords:** ncRNA, metabolic reprogramming, circRNAs, microRNA, lncRNA

## Abstract

Cancer cells are primarily characterized by uncontrolled proliferation, which is closely linked to an increased energy demand. According to this, cancer cells preferentially utilize the glycolytic pathway for energy production. Metabolic regulation in cancer occurs at multiple levels, including post-translational modifications, which are rapid and energy-efficient, as they do not require enzyme degradation. In this context, non-coding RNA-mediated control plays a crucial role, as these small RNAs can directly modulate the mRNAs of key metabolic enzymes or induce epigenetic modulations, ultimately inducing alterations in cancer metabolism. In this mini-review, we summarize how non-coding RNAs (ncRNAs), including microRNAs, long non-coding RNAs (lncRNAs), and circular RNAs (circRNAs), modulate core metabolic pathways, including glycolysis, mitochondrial respiration, lipid metabolism, and amino acid biosynthesis, in cancer. We also highlight recent findings that uncover novel regulatory mechanisms and propose that ncRNAs serve as dynamic integrators of metabolic and oncogenic signals. Understanding these interactions may open new avenues for biomarker identification and ncRNAs-targeted therapies development.

## Graphical Abstract

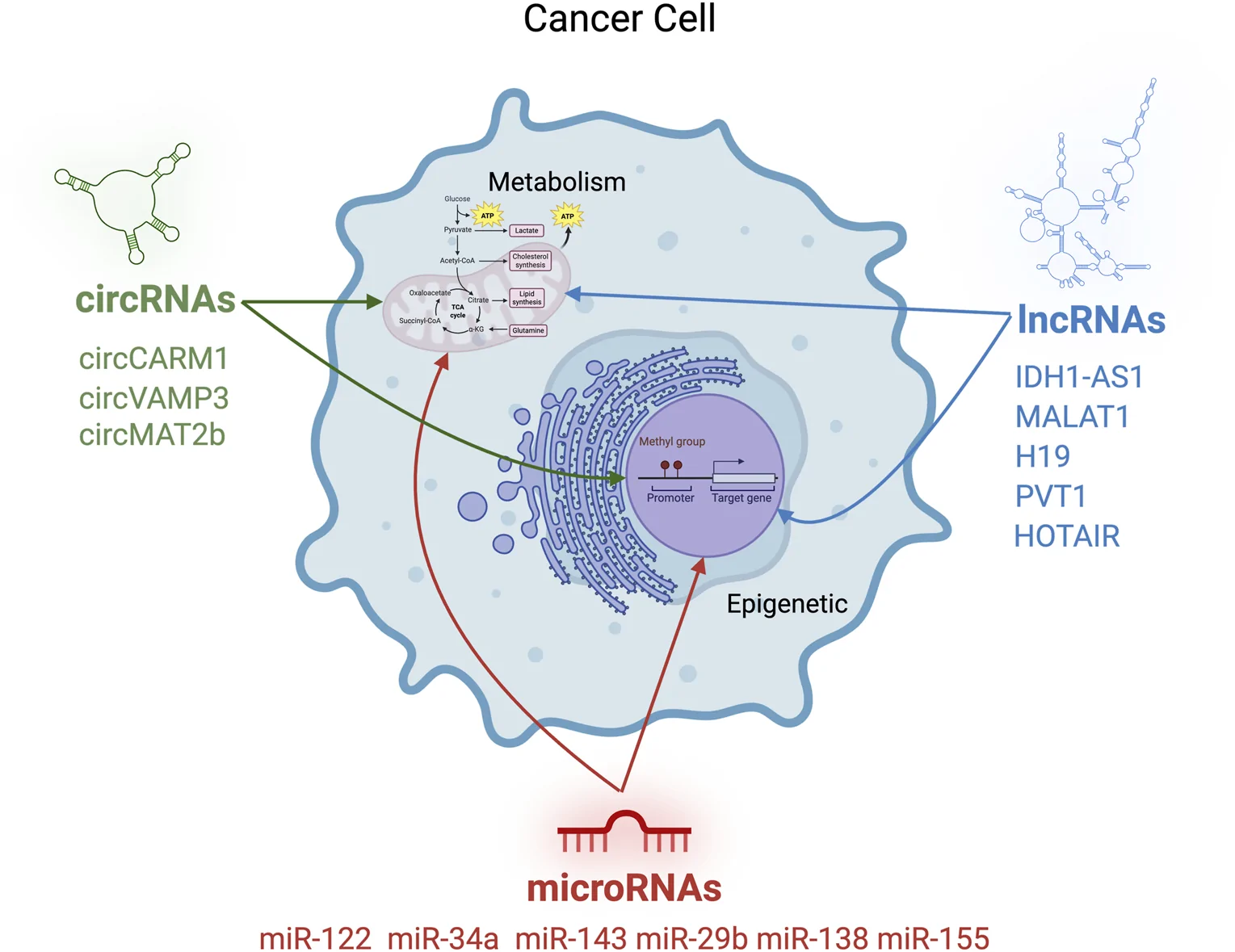

## 1 Introduction

Cancer metabolism is characterized by dramatic shifts in nutrient utilization, including enhanced aerobic glycolysis (the Warburg effect), increased lipid biosynthesis, and reprogramming of amino acid and mitochondrial metabolism (Mathew et al., 2024). While transcriptional regulators such as MYC and HIF-1α have been well studied in this context (Dong et al., 2020; Taneja et al., 2024), accumulating evidence reveals that non-coding RNAs (ncRNAs) are also central to this process. These molecules do not code for proteins but exert regulatory functions through RNA-RNA, RNA-DNA, or RNA-protein interactions. Although approximately 75% of the human genome is transcribed into RNA, only about 3% of these transcripts encode proteins. The majority of non-protein-coding transcripts correspond to ncRNAs (Yan and Bu, 2021; Zong et al., 2023).

NcRNAs can be broadly classified into three main categories. The first group includes housekeeping ncRNAs, such as tRNAs, rRNAs, snRNAs, and snoRNAs, which play essential structural and functional roles in cell biology. These RNAs are constitutively expressed across all cell types. The second group consists of small ncRNAs, including microRNAs (miRNAs) which will be the focus of this review small interfering RNAs (siRNAs), PIWI-interacting RNAs (piRNAs), and small Cajal body-specific RNAs (scaRNAs). These molecules are widely involved in gene expression regulation at multiple levels, acting both on chromatin and on specific mRNA targets. The third group comprises long ncRNAs (lncRNAs), which include long intergenic non-coding RNAs (lincRNAs) and circular RNAs (circRNAs), discussed below, as well as enhancer RNAs (eRNAs) and natural antisense transcripts (NATs) (Eldakhakhny et al., 2024; Fu, 2014). ncRNAs regulate gene expression through a variety of mechanisms, which are applied across multiple cellular pathways. These include pathways involved in differentiation (Mishra et al., 2023), proliferation, apoptosis (Tüncel et al., 2022; Wu et al., 2024), inflammation (Zhang et al., 2024), and, notably, metabolism. miRNAs have emerged as key regulators of metabolic processes, with growing evidence supporting the functional specificity of distinct miRNA families in targeting defined metabolic pathways. Several miRNA clusters are found to be overexpressed in various tumour types, contributing to oncogenesis by downregulating the expression of specific tumour suppressor proteins. This subset of miRNAs modulates mitochondrial metabolism, acting as oncogenic drivers through the suppression of oxidative phosphorylation (Thirunavukkarasu et al., 2024). Conversely, certain miRNAs function as tumor suppressors by downregulating the expression of key factors involved in glycolytic metabolism, such as glucose transporters (GLUTs). These tumor-suppressive miRNAs are frequently downregulated in cancer, thereby facilitating the metabolic reprogramming that supports tumor growth and progression (Safi et al., 2023). LncRNAs exert their regulatory functions through interactions with metabolic enzymes, modulating protein levels via mechanisms that include post-translational modifications, epigenetic alterations, lncRNA-mRNA binding that interferes with translation and miRNAs sponging. Through these diverse molecular mechanisms, lncRNAs can function either as oncogenic drivers or as tumor suppressors, depending on the context (Safi et al., 2023; Tan et al., 2021; Alluri et al., 2025). In addition, circRNAs also play a role in regulating metabolic reprogramming in cancer. As with other non-coding RNAs, their pro- or anti-tumorigenic functions are highly context-dependent and vary across different cancer types. A key feature of circRNAs is their ability to mediate long-distance cellular communication, partly due to their presence in extracellular vesicles such as exosomes (Liu et al., 2022). In this context, circRNAs have been primarily investigated for their involvement in the metastatic spread to distant peripheral sites from the primary tumor, as well as for their potential role as biomarkers for both diagnostic and prognostic purposes. Given the vast diversity of ncRNAs and, more importantly, their context-dependent roles across different tumor types, this review aims to summarize and integrate current knowledge on the involvement of ncRNAs, specifically, miRNAs, lncRNAs, and circRNAs, in the regulation of cancer metabolism.

## 2 MicroRNAs: small regulators with broad impact

MiRNAs are a class of ncRNAs that regulate gene expression post-transcriptionally, acting after mRNA synthesis and before its translation into protein. MiRNAs exert their function by recognizing and binding, with high specificity, to complementary sequences typically located in the 3′ untranslated region (3′ UTR) of target mRNAs. This interaction inhibits translation by promoting deadenylation and decapping, ultimately leading to mRNA degradation (O’Brien et al., 2018). Additionally, miRNAs have been shown to bind other regions of the mRNA, including the 5′ UTR and coding sequences, which can also suppress translation (Xu et al., 2014). Interestingly, miRNA binding to promoter regions has also been associated with upregulation of gene expression, indicating that miRNAs may exert both repressive and activating regulatory functions depending on the binding context (Dharap et al., 2013).

A growing body of evidence links the expression of specific miRNA clusters to metabolic reprogramming in cancer cells. For instance, miR-122, often downregulated in hepatocellular carcinoma, suppresses glycolysis and lipid metabolism by targeting pyruvate kinase and FASN (Chun, 2022). Similarly, miR-34a and miR-143 regulate key enzymes in glucose metabolism and the TCA cycle (Zhao et al., 2016; Zhang and Schulze, 2016). These miRNAs often function as tumor suppressors and are dysregulated in multiple cancer types. Beyond their direct involvement in the post-transcriptional regulation of metabolism-related mRNAs, microRNAs also contribute to epigenetic regulation mechanisms, a key aspect explored in this review.

In addition to being regulated by epigenetic mechanisms, miRNAs themselves can exert epigenetic effects, primarily by modulating the expression of key epigenetic enzymes such as DNMTs, TETs, HDACs, and EZH2 (Sato et al., 2011). Due to this specific regulatory activity, such miRNAs are referred to as epi-miRNAs; several of them are included in Table 1. Among them, miR-29b, part of the miR-29 family targets both DNMTs and TET enzymes. The downregulation of miR-29b leads to increased expression of DNMT3A, which in turn promotes the silencing of the tumor suppressor gene PTEN, a negative regulator of glycolysis and a positive regulator of energy expenditure (Yao et al., 2019). MiR-138 has been shown to downregulate the histone demethylase Lysine demethylase 5B (KDM5b), down-regulating key genes of lipid metabolism (including FASN and ACLY), thereby suppressing the proliferation and migration of breast cancer cells (Denis et al., 2016). KDM5b, indeed, is overexpressed in multiple cancer types, and elevated expression levels of this enzyme are associated with decreased survival (He et al., 2022). KDM5b has emerged as a promoter of cancer-associated lipid metabolic reprogramming, and its downregulation has been proposed as a potential therapeutic strategy in various tumors, including breast cancer, liver cancer, and pancreatic ductal adenocarcinoma (Jose et al., 2020; Zhang et al., 2019). MiR-137 is another microRNA implicated in epigenetic regulation associated with metabolic reprogramming. Among its targets is lysine (K)-specific demethylase 1A (LSD1), an epigenetic modifier known to exert widespread effects on genomic methylation (Yao et al., 2019). LSD1 has been reported to enhance glucose uptake, promoting glycolysis over mitochondrial metabolism and thereby contributing to the Warburg effect. Among the underlying mechanisms, LSD1 stabilizes HIF-1α, facilitating the expression of glycolytic genes such as hexokinase. In parallel, LSD1 promotes the degradation of key regulators of mitochondrial biogenesis, including PGC-1α. Additionally, LSD1 upregulates c-MYC, a well-known oncogenic factor that further supports glycolysis and tumor cell proliferation (Cheng et al., 2025). Epi-miRNAs have also been associated with the ability of cancer cells to adapt to stress conditions, such as hypoxia, which is characteristic of the inner regions of solid tumors. Notably, miR-155 has been shown to affect H3K36me2 expression under hypoxic conditions by repressing the histone lysine demethylase KDM2a, thereby limiting its activity. The miR-155–KDM2a interaction is essential for preventing excessive reactive oxygen species (ROS) production and for maintaining proper regulation of mitochondrial gene expression (Nakagawa et al., 2024). Epi-miRNAs have also been shown to play a key role in regulating the metabolism of immune cells, which are typically present in the tumor microenvironment. In these cells, metabolic pathways are closely linked to polarization and differentiation into either a pro-inflammatory, glycolytic phenotype, or an anti-inflammatory, oxidative phenotype. In this context, a representative example is epi-miR-143, which targets DNA methyltransferase 3A (DNMT3A). This miRNA can modulate the expression of pro-glycolytic genes, such as hexokinase and GLUT1, thereby influencing the metabolic programming of immune cells (Alshahrani et al., 2022). Based on this, the interest toward epi-miRNAs is growing since targeting a single miRNA can influence a broad network of downstream targets. In the case of epi-miRNAs, this effect is further amplified, as epigenetic regulators can induce widespread changes across the entire genome.

**TABLE 1:** Non-coding RNAs involved in metabolic regulation in cancer.

ncRNA	Type	Targets	Metabolic pathway	Cancer/Context	Functional outcome	Ref.
miR-122	microRNA	PKM2 and FASN	Glycolysis; lipid metabolism	Hepatocellular carcinoma	Suppresses glycolysis and lipid metabolism	Chun (2022)
miR-34a	microRNA	TCA cycle	Glycolysis; TCA	Multiple cancers	Tumor-suppressive control of metabolic enzymes	Zhao et al. (2016), Zhang and Schulze (2016)
miR-143	microRNA	TDNMT3A	Glycolysis	Immune cells in TME	Reprograms immune-cell metabolism (pro-/anti-glycolytic)	Zhao et al. (2016), Zhang and Schulze (2016)
miR-29b	microRNA	DNMTs and TETs	Glycolysis regulation	Multiple cancers	Promotes glycolytic shift via PTEN silencing when downregulated	Yao et al. (2019)
miR-138	microRNA	KDM5B FASN, ACLY	Lipid metabolism	Breast cancer	Suppresses proliferation/migration via lipid-gene downregulation	Denis et al. (2016)
miR-137	microRNA	LSD1	Glycolysis vs. mitochondrial metabolism	Multiple cancers	LSD1 axis favors glycolysis; miR-137 counters this	Yao et al. (2019)
miR-155	microRNA	KDM2A	Mitochondrial/oxidative metabolism; hypoxia response	Hypoxic tumor regions	Supports adaptation to hypoxia; controls ROS	Nakagawa et al. (2024), Abtin et al. (2018)
IDH1-AS1	lncRNA	IDH1	Glycolysis (attenuation)	Multiple cancers	Reduces glycolysis; overexpression suppresses proliferation	Xiang et al. (2018), Liu et al. (2018)
HOTAIR	lncRNA	UPP1	Uridine bypass; lipid/metabolic gene control	Colorectal cancer; multiple cancers	Promotes proliferation/invasion; therapy resistance	Hajjari and Salavaty (2015), Cantile et al. (2024)
MALAT1	lncRNA	mTORC1,4EBP1,SRSF1 TCF7L2	Lipid metabolism; glycolysis	Hepatic lipid accumulation; cancer (general)	Increases triglyceride/lipid handling; promotes glucose metabolism	Cantile et al. (2024), Malakar et al. (2019)
H19	lncRNA	miR-19a-3p	Glycolysis/lactate production	Gastric cancer	Enhances glycolysis and tumor growth	Zhong et al. (2021), Chen et al. (2023)
PVT1	lncRNA	MYC and STAT3	Glycolysis; lipid metabolism	Multiple cancers; adipogenesis	Upregulates glycolytic enzymes; promotes lipid accumulation	Wu et al. (2023), Jin et al. (2019)
circVAMP3	circRNA	LDHA	Glycolysis	Renal cell carcinoma	Promotes aerobic glycolysis and proliferation	Li et al. (2022)
circMAT2B	circRNA	miR-338-3p	Glycolysis	Hepatocellular carcinoma	Enhances glycolysis and malignancy	Liao et al. (2022)
circCARM1	circRNA	PFKFB2	Glycolysis regulation	Breast cancer (spheroids/exosomes)	Reprograms cell metabolism via PFKFB2	Liu et al. (2022)
snRNAs	snRNA	-	Metabolic programming via differentiation	Adipose tissue/cancer metabolism	Influence metabolic responses through differentiation control	Yang et al. (2025)
snoRNAs	snoRNA	-	Lipid metabolism/metabolic stress	Steatohepatitis; metabolic reprogramming	Contribute to lipid-induced cellular stress and reprogramming	Li et al. (2025)

### 2.1 Long non-coding RNAs: The mastermind of metabolic adaptation

Emerging evidence indicates that lncRNAs play a central role in orchestrating and reinforcing cancer cells’ metabolic reprogramming. LncRNAs are transcripts longer than 500 nucleotides that lack coding potential but exert broad regulatory effects through diverse mechanisms (Palma et al., 2025). Functionally, they can act as guides, recruiting chromatin-modifying complexes to specific loci; scaffolds, assembling multiprotein complexes; decoys, sequestering transcription factors or miRNAs; and signals, reflecting the activity of distinct transcriptional programs (Mangraviti and De Windt, 2022). These categories often overlap, and many lncRNAs operate through multiple mechanisms simultaneously. In cancer, lncRNAs are emerging as pivotal integrators of epigenetic remodeling and metabolic reprogramming, enabling tumor cells to dynamically adapt to nutrient availability and therapeutic pressure.

Among the best characterized oncogenic lncRNAs is IDH1-AS1, which functions as a metabolic regulator by enhancing IDH1 homodimerization (Wu et al., 2020). This activity increases α-ketoglutarate levels and reduces ROS, resulting in downregulation of HIF-1α and attenuation of aerobic glycolysis. However, in cancer cells under normoxic conditions, the oncogene c-MYC transcriptionally represses IDH1-AS1, thereby maintaining HIF-1α activity and sustaining glycolytic metabolism. Experimentally, IDH1-AS1 overexpression suppresses tumor cell proliferation, whereas its knockdown promotes proliferation and tumor growth in xenograft models, highlighting the therapeutic potential of restoring IDH1-AS1 expression (Xiang et al., 2018; Liu et al., 2018).

HOTAIR exemplifies the guide function. It is significantly overexpressed in numerous cancers, including colorectal, breast, brain, lung, pancreatic, and gastric cancers (Hajjari and Salavaty, 2015). By predominantly recruiting PRC2, HOTAIR regulates a wide range of targets in trans, modulating both gene expression and alternative splicing, thereby influencing adipocyte development and human fat distribution (Kuo et al., 2022a). In addition, HOTAIR promotes H3K27me3 deposition and transcriptional silencing of genes enriched in nervous system developmental pathways and lipid metabolism (Kuo et al., 2022b). Its epigenetic activity has been implicated in resistance to chemotherapy, linking metabolic regulation to therapy adaptation. In colorectal cancer cells, HOTAIR drives proliferation and invasion by upregulating the metabolic enzyme UPP1 via recruitment of the histone methyltransferase EZH2, which deposits H3K27me3 on the UPP1 promoter. This HOTAIR–EZH2–UPP1 axis enables cancer cells to exploit uridine as an alternative energy source, a process known as the uridine bypass, thereby sustaining tumor growth under glucose limitation (Chen et al., 2024). Moreover, by coupling metabolic regulation with autophagy control, HOTAIR reduces the efficacy of radiotherapy and chemotherapy (Cantile et al., 2024).

Another well-characterized oncogenic lncRNA is MALAT1, whose upregulation contributes to hepatic lipid accumulation in response to oleic acid. MALAT1 regulates a network of lipid metabolism–related genes (Khalifa et al., 2025), particularly those involved in triglyceride synthesis and lipid handling, rather than canonical regulators such as SREBP-1 or PPARγ. These findings suggest that MALAT1 modulates both transcriptional and post-transcriptional layers of lipid metabolism. In cancer, MALAT1 promotes glucose metabolism through the mTORC1–4EBP1 signaling axis, where it increases SRSF1 expression to enhance translation of the transcription factor TCF7L2 (Malakar et al., 2019).

The imprinted lncRNA H19 illustrates the interplay between metabolism, chromatin state, and environmental cues. H19 is overexpressed in several cancers, including gastric cancer, where it correlates with poor prognosis. In normal physiology, H19 regulates processes such as neurogenesis and neural stem cell differentiation (Zhong et al., 2021), but it can also promote glucose consumption and lactate production by modulating metabolic enzymes. In gastric cancer, H19 enhances glycolysis and tumor growth by sponging miR-19a-3p, thereby upregulating the glycolytic enzyme PGK1. Silencing H19 reduces glycolysis, proliferation, and tumor size *in vivo*, confirming its oncogenic metabolic role (Chen et al., 2023).

Another important lncRNA reinforcing this epigenetic–metabolic axis is PVT1. This lncRNA is a critical regulator of differentiation and development (Wu et al., 2023). In the context of adipogenesis, PVT1 is upregulated during 3T3-L1 differentiation and in adipose tissue, where it promotes lipid accumulation by activating PPARγ, C/EBPα, and aP2, likely through interaction with STAT3, while enhancing fatty acid synthesis and inhibiting oxidation (Archambault et al., 2021). In cancer, PVT1 is frequently co-amplified with MYC. PVT1 stabilizes MYC protein, promotes expression of glycolytic enzymes, and thereby enhances tumor energy metabolism (Jin et al., 2019).

## 3 The new player on the board in metabolic regulation: CirRNA, SnoRNAs, tiRNAs

Although miRNAs and lncRNAs are now broadly recognized as major regulators of metabolic function and cancer progression, recent years have seen growing attention to additional classes of ncRNAs with similar functions. Among the most relevant are circRNAs, a large class of single-stranded RNA molecules that form covalently closed continuous loops with their 5′ and 3′ ends joined together (Awas et al., 2018). This unique structure lacks the typical termini of linear RNAs and often does not include a poly(A) tail. Mechanistically, circRNAs are generated through back-splicing, a process in which a downstream 5′ splice site joins to an upstream 3′ splice site of the same or different exon(s) within the pre-mRNA, resulting in a circular transcript. Important examples include circVAMP3, which promotes aerobic glycolysis and proliferation by regulating LDHA in renal cell carcinoma (Li et al., 2022), and circMAT2B, which similarly promotes glycolysis and malignancy in hepatocellular carcinoma through the miR-338-3p/PKM2 axis (Ferrera et al., 2025). Other examples of circRNAs found to be closely related to metabolic reprogramming are circMAT2B, circSPECC1, circC3P1 and circRPN2 (Liao et al., 2022).

Despite the fact that miRNAs, circRNAs, and lncRNAs are still considered the three major classes of ncRNAs in cancer metabolism, additional and less clearly defined RNA species are emerging as relevant regulators. Examples include small nuclear RNAs (snRNAs), which are non-coding RNAs of approximately 100–300 nucleotides localized in the nucleus and functioning as essential components of the spliceosome (Webster and Ghalei, 2023), and small nucleolar RNAs (snoRNAs), small RNAs of 60–300 nucleotides mainly located in the nucleolus that guide chemical modifications of other RNAs, particularly ribosomal RNAs (rRNAs), transfer RNAs (tRNAs), and snRNAs (Bratkovič et al., 2020). In particular, snoRNAs can impact various metabolic pathways, including the p53 pathway, which acts as a key regulator of tumor oxidative metabolism. They also influence the PI3K/AKT pathway and the MAPK signaling cascade, which is itself associated with the regulation of oxidative stress and autophagy (Li et al., 2025; Desideri et al., 2025). Compelling evidence suggests that snoRNAs contribute to lipid-induced cellular stress and metabolic reprogramming, including pathological processes such as steatohepatitis, whereas snRNAs have been implicated in the control of proper adipose tissue differentiation, thereby strongly influencing metabolic responses in cancer (Yang et al., 2025).

## 4 Discussion

The interplay between ncRNAs, epigenetic remodeling, and metabolic reprogramming defines a multilayered regulatory framework that grants cancer cells remarkable plasticity. MiRNAs, lncRNAs, and circRNAs converge on chromatin modifiers and metabolic enzymes to sustain glycolysis, repress mitochondrial respiration, and diversify lipid and amino acid metabolism. These molecules act within feedback loops integrating nutrient availability, metabolite levels, and chromatin state, allowing tumors to adapt to environmental and therapeutic pressures (Zong et al., 2023; Shin et al., 2025; Wang et al., 2022). For example, both miR-199a and miR-122 reduce the expression of pyruvate kinase M2 (PKM2) and are therefore downregulated under hypoxic conditions, which are common in cancer. This downregulation promotes glycolysis and supports the Warburg effect (Liao et al., 2022). In addition, hypoxia leads to the downregulation of miR-125a, which in turn results in the upregulation of hexokinase 2 (HK2), a key rate-limiting enzyme in the glycolytic pathway (Jin et al., 2017).

Despite progress, several challenges remain. ncRNAs often display context-dependent roles, similarly, circRNAs and lncRNAs that drive glycolysis in one setting may promote oxidative metabolism in another. Such dualities highlight the need for systematic studies across tumor types, ideally employing single-cell and spatial transcriptomics to capture heterogeneity. Such heterogeneity is associated with the fact that ncRNAs can exert their effects at multiple levels, and the response may depend on the basal expression level of specific genes, including those involved in the expression of metabolic enzymes. Many transcription factors altered in cancer are involved in metabolic regulation and are themselves regulated by ncRNAs, including the previously mentioned MYC. Therefore, the role of a specific ncRNA also depends on the basal expression of these factors, that could be different even the same tumor mass (Alfar et al., 2021). Another unresolved issue is causality within ncRNA–epigenetic–metabolic circuits: it is often unclear whether an ncRNA initiates remodeling or is induced by metabolic stress. Addressing this will require integrated approaches combining genetic perturbation, metabolomics, and chromatin profiling.

From a translational perspective, targeting ncRNAs remains promising but challenging. Antisense oligonucleotides, siRNAs, and CRISPR-based tools show potential, but specificity and delivery remain hurdles. Small molecules that disrupt lncRNA–protein interactions or circRNA biogenesis are emerging, while the stability of circRNAs and tissue specificity of many lncRNAs render them attractive biomarkers (Ruzi et al., 2025). In this regard, high-throughput screening of chemical libraries against RNA targets has led to the identification of promising lead compounds with high binding affinities. In this context, several small molecules have been identified capable of interfering with miRNA biogenesis (Ruzi et al., 2025; Syed et al., 2024). For example, oleuropein has been shown to reduce proliferation and induce apoptosis in breast cancer by downregulating miR-21 and miR-155, both oncomiRs which can favor glycolysis (Abtin et al., 2018; Hashemi et al., 2023). Among the different strategies that have been developed to suppress oncomiRs, there are anti-miRs (AMOs), miRNA sponges, miRNA masking, and small molecule inhibitors (SMIRs). AMOs are chemically modified synthetic antisense oligonucleotides of simple design and ease of synthesis. The main limitation is that their inhibitory effects are often transient due to dilution during cell division. MiRNA sponges are constructs containing multiple tandem binding sites complementary to a specific miRNA, effectively sequestering it and preventing interaction with its natural targets. MiRNA masks are single-stranded antisense oligonucleotides that bind to specific miRNA recognition elements on target mRNAs. Finally, SMIRs act at various stages of miRNA biogenesis (Arghiani and Shah, 2021).

Looking forward, integrating multi-omics with artificial intelligence and systems biology will be essential to map ncRNA–epigenetic–metabolic networks. Ultimately, resolving their mechanistic complexity may transform ncRNA-driven plasticity from an obstacle into a therapeutic vulnerability, advancing biomarker discovery and precision oncology.
